# Supplementary material for: Screen for mitochondrial DNA copy number maintenance genes reveals essential role for ATP synthase
Source: Mol Syst Biol. 2014 Jul 1;10(6):734. doi: 10.15252/msb.20145117 (PMC4265055; doi:10.15252/msb.20145117)
Supplement: Supplementary file 1 — Supplementary Figure S1 [file msb0010-0734-sd1.pdf]

0. Experimental references

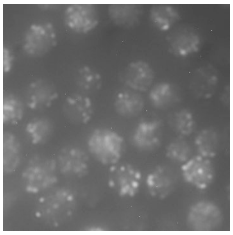
Negative control (GFP)

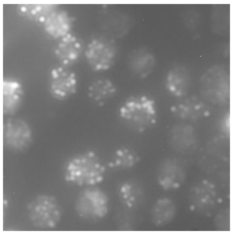
Negative control (No RNAi)

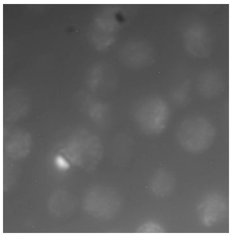
Positive control (Polg)

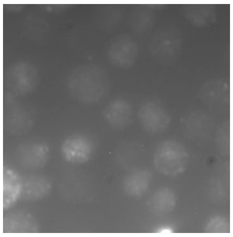
Negative control (mtDNA helicase)

1. mtDNA replication and transcription

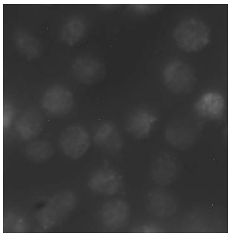
CG3910

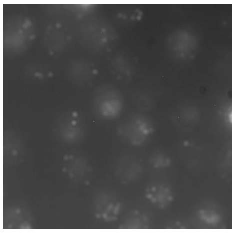
CG4217

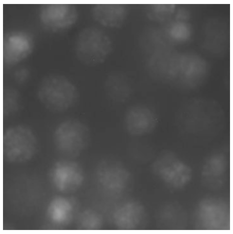
CG4337

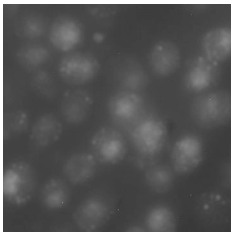
CG4644

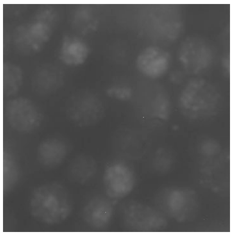
CG5924

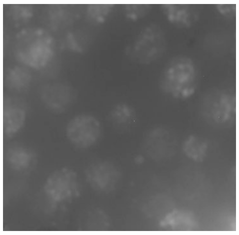
CG6815

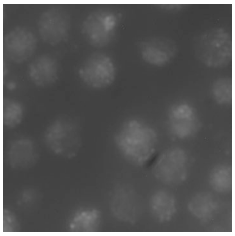
CG7175

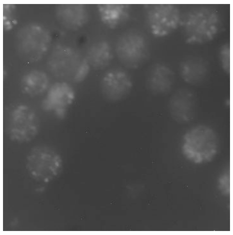
CG8729

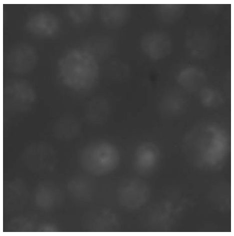
CG8987

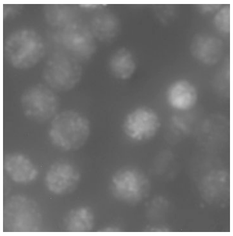
CG18124

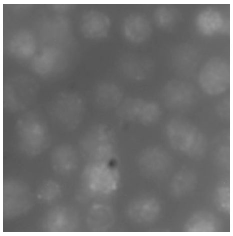
CG33650

2. Cytosolic translation

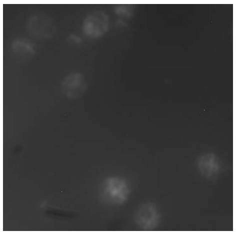
CG1821

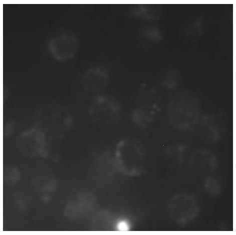
CG3203

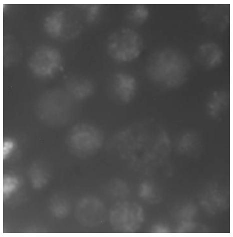
CG3751

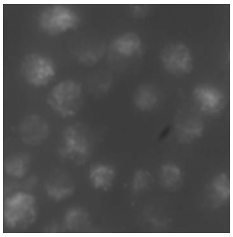
CG3922

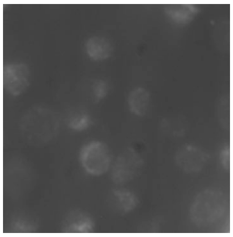
CG3997

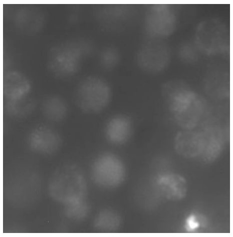
CG4111

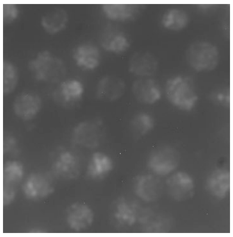
CG4759

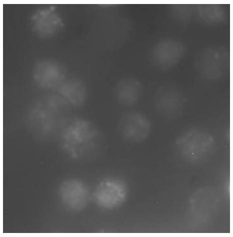
CG7283

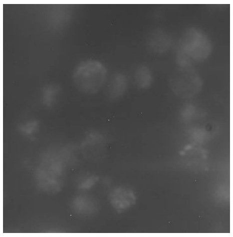
CG7490

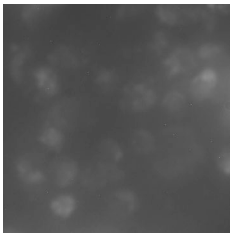
CG7622

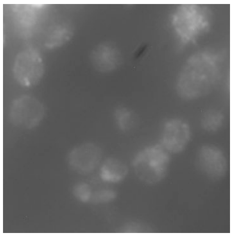
CG7726

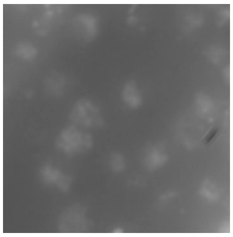
CG8922

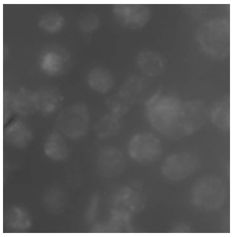

CG9282

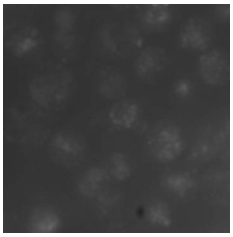

CG9677

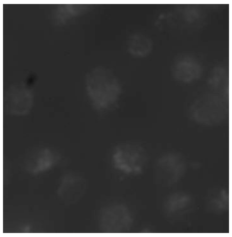

CG11522

3. Proteasome

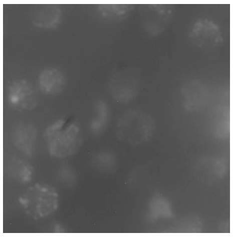

CG1341

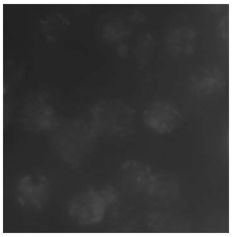

CG5266

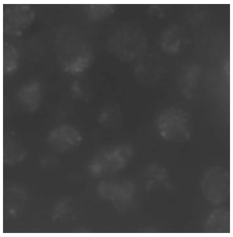

CG9324

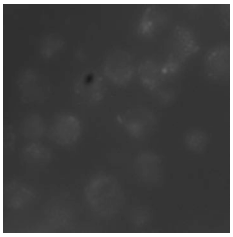

CG9327

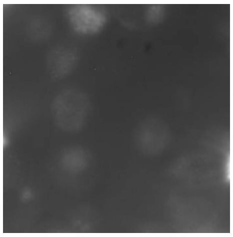

CG10149

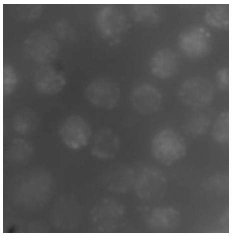

CG16916

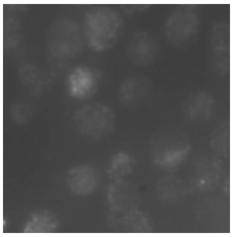

CG18174

4. ATP synthase

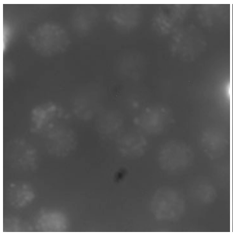

CG2968

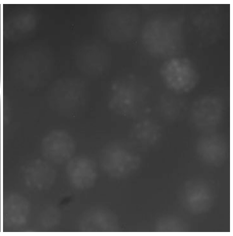

CG3321

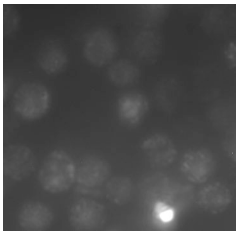

CG4307

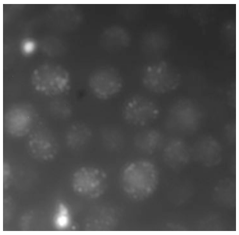

CG4412

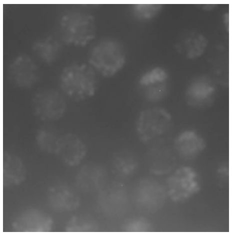

CG6015

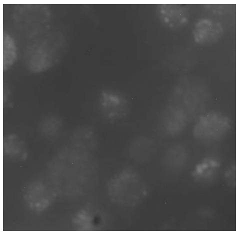

CG7610

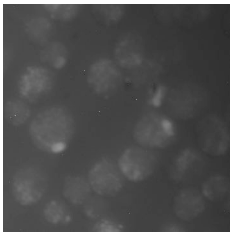

CG6030

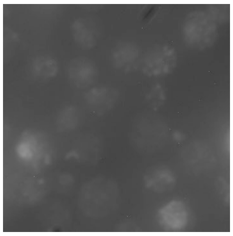

CG8189

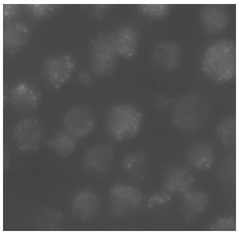

CG11154

5. Mitochondrial biogenesis or dynamics

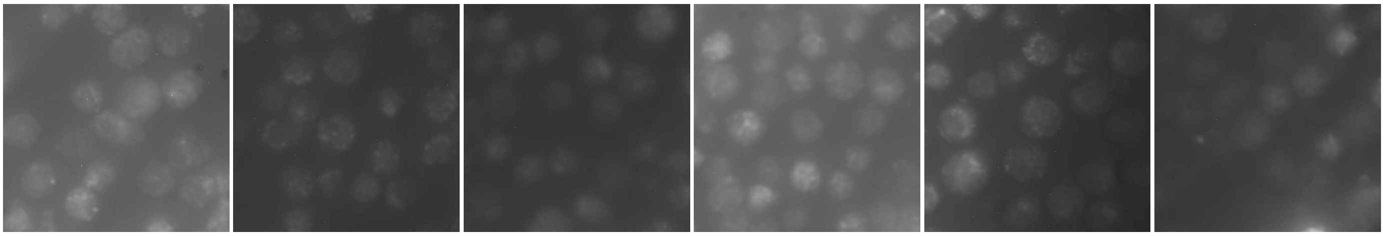

CG3114 CG6338 CG6512 CG8479 CG9809 CG14981

6. Nuclear gene expression

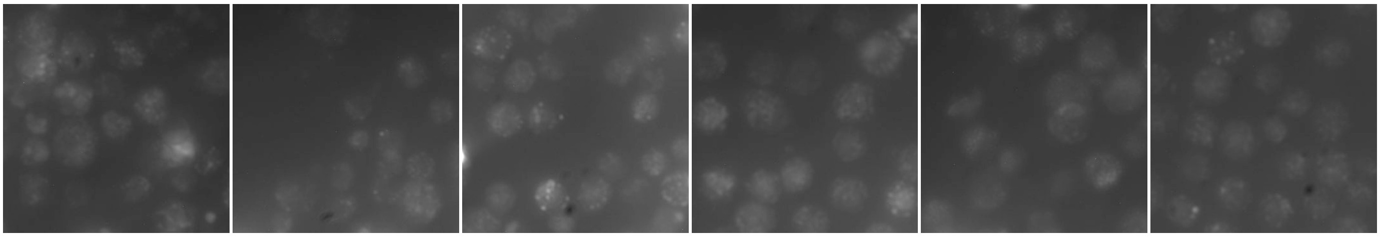

CG1057 CG1554 CG1810 CG1874 CG2163 CG3162

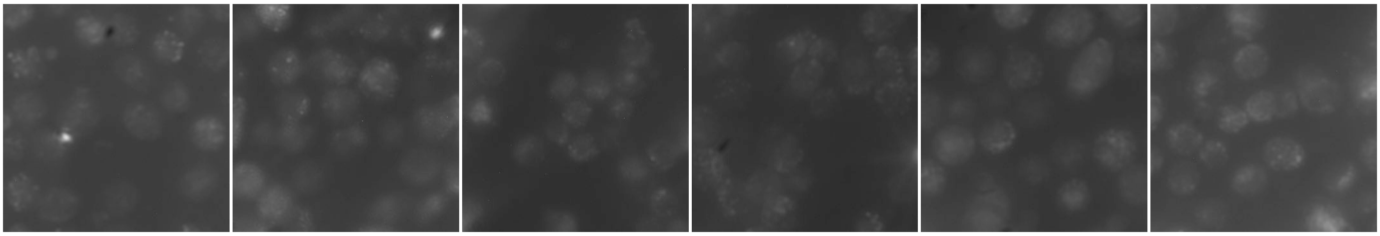

CG3675 CG6525 CG7626 CG9591 CG9748 CG10955

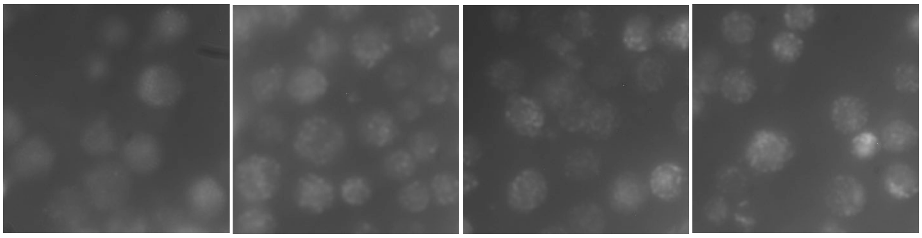

CG11990 CG17183 CG17358 CG17603

7. Other or unknown

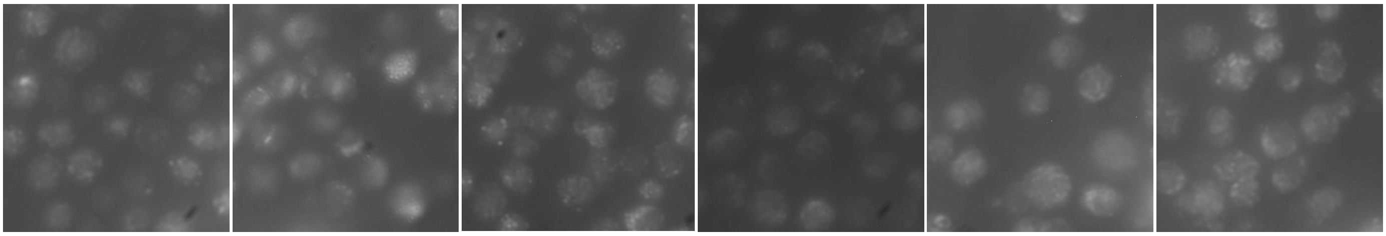

CG3539 CG4268 CG5794 CG6413 CG7368 CG8021

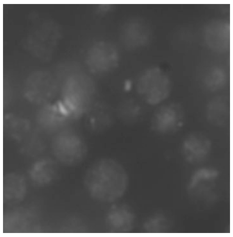
CG9007

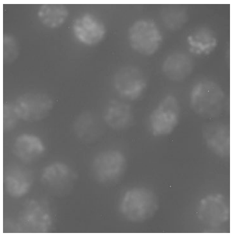
CG9397-H

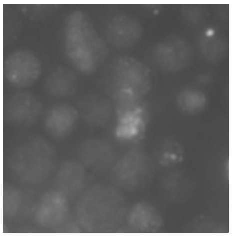
CG9797

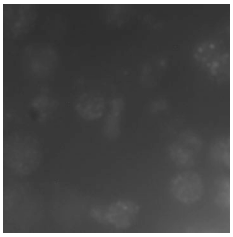
CG10042

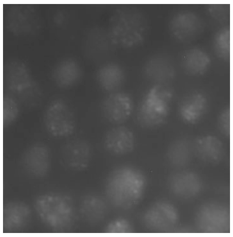
CG10144

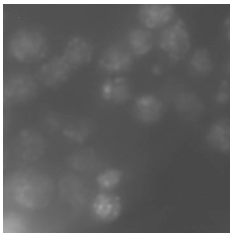
CG10395

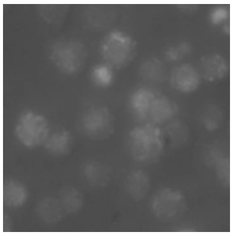
CG10582

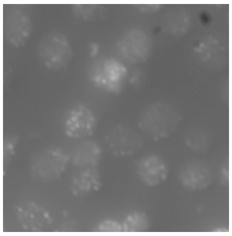
CG10582-C

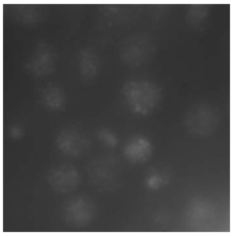
CG12242

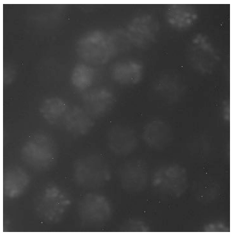
CG13203-C

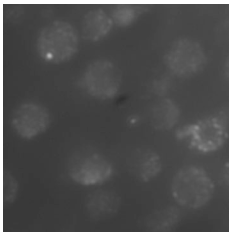
CG13779

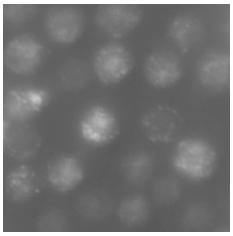
CG14084

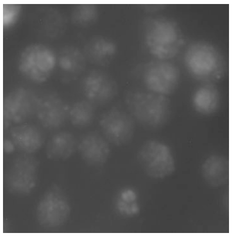
CG14247

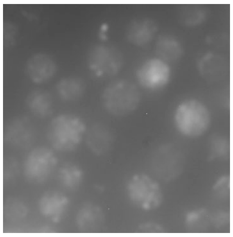
CG14634

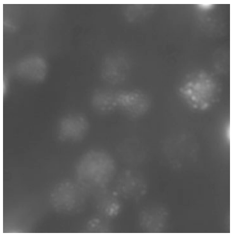
CG15231

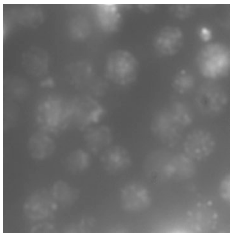
CG15343

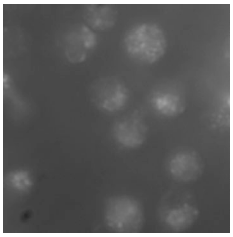
CG15793

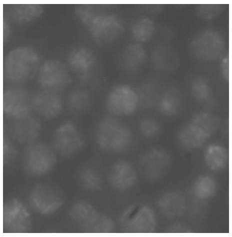
CG17077

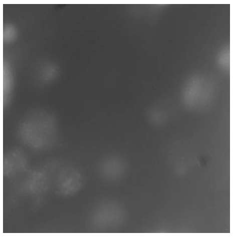
CG31258

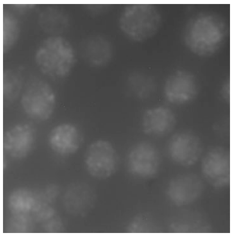
CG31079

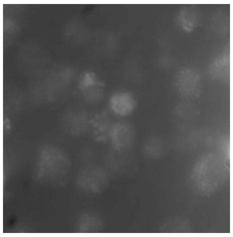
CG32085

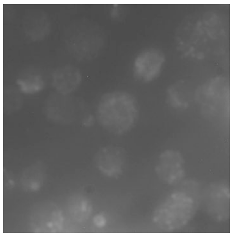
CG32561,2

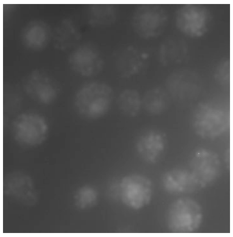
CG32570

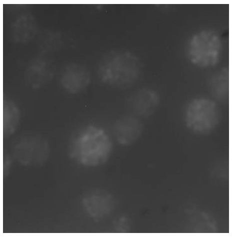
CG32652

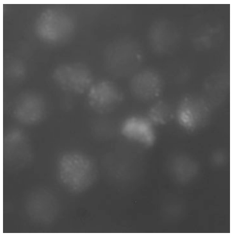
CG34415

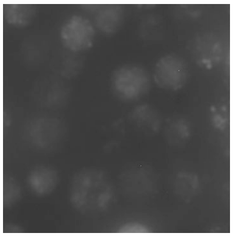
CG33546

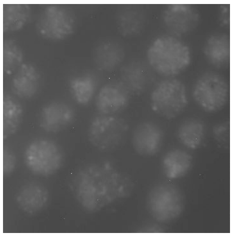
CG42666-D

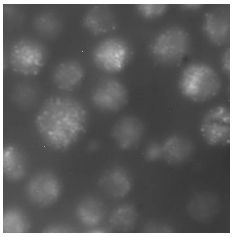
CG42666-G

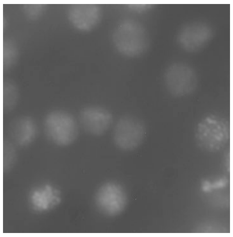
CG42281

8. Negative (examples)

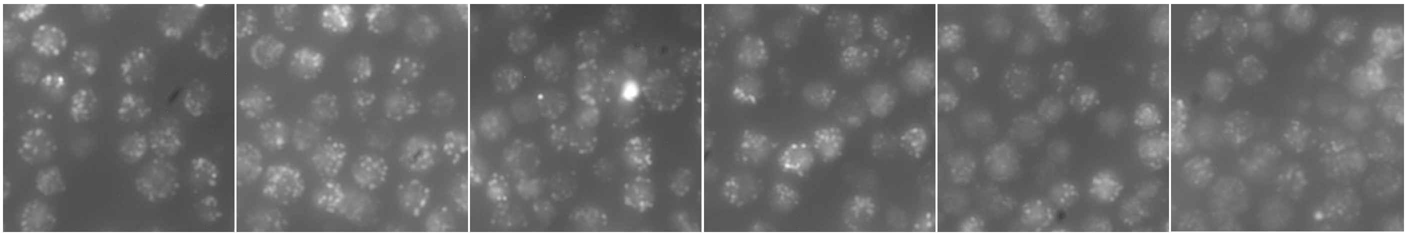

CG1021 CG1048 CG1193 CG3758 CG4120 CG5905

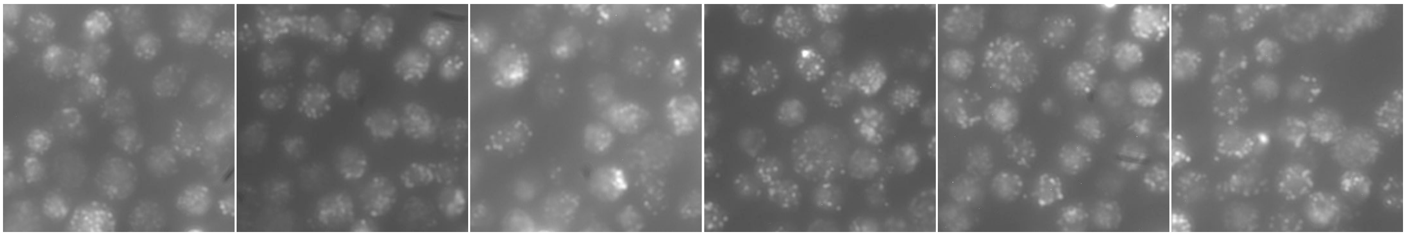

CG5728 CG5818 CG8532 CG32465 CG32602 CG33297

Figure S1

Screening of *Drosophila* dsRNA library by PicoGreen nucleoid fluorescence in S2 cells

Supplementary data for Fig. 1. Micrographs of S2 cells stained with PicoGreen, following 5 d of treatment with the dsRNA indicated. A blank (no RNAi) and dsRNA against GFP were used as negative controls in every plate screened. dsRNAs directed against Polg (tamas, CG8987) and, during rescreening, also mtDNA helicase (CG5924) were used as positive controls. Both were detected in the blinded screen as positives, as indicated. Images of positives are grouped according to the gene categories shown in Table 1. The dsRNA against CG32561 also targets the closely related gene CG32562. dsRNA targeted against specific splice isoforms of CG10582, CG13203 and CG42666 mRNAs are indicated accordingly.
