# Supplementary material for: Screen for mitochondrial DNA copy number maintenance genes reveals essential role for ATP synthase
Source: Mol Syst Biol. 2014 Jul 1;10(6):734. doi: 10.15252/msb.20145117 (PMC4265055; doi:10.15252/msb.20145117)
Supplement: Supplementary file 2 — Supplementary Figure S2 [file msb0010-0734-sd2.pdf]

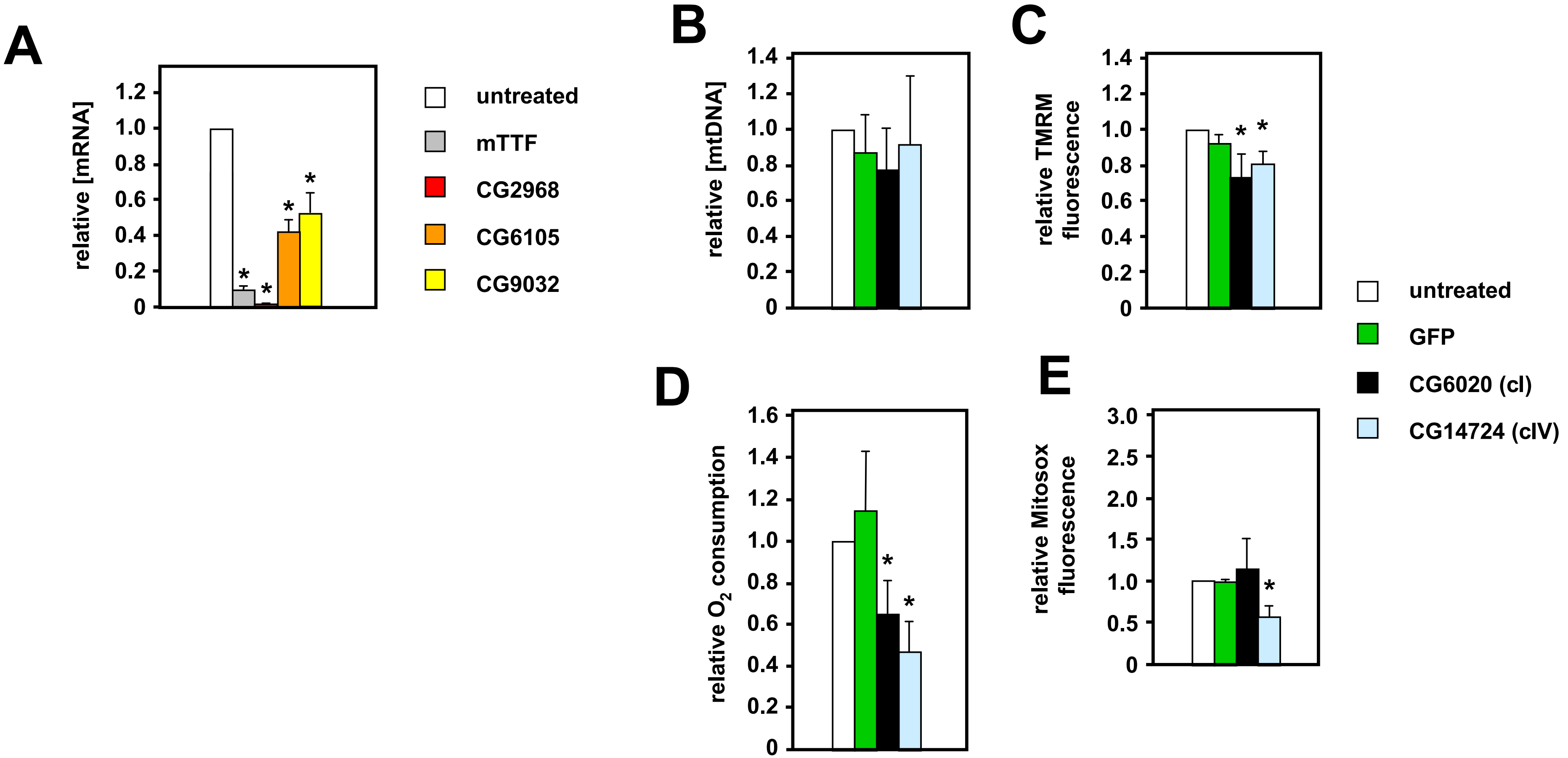

Figure S2, Fukuoh et al

Figure S2

Knockdown of subunits of ATP synthase

(A) Relative levels of the indicated mRNAs, normalized to values from untreated cells grown in parallel, following 5 d of treatment with dsRNA against the cognate gene. (Note that the GFP control would not be meaningful in this case, since the cells have no GFP gene). dsRNAs against mTTF and CG2968 produced >90% knockdown at RNA level, whereas those against CG6105 and CG9032 produced much more modest effects. Means + SD from three experiments, each conducted in triplicate. (B-E) Effects of knockdown of subunits of cI (CG6020, NDUFA9) or cIV (CG14724, COX5A). (B) Relative mtDNA level, (C) TMRM fluorescence, (D) whole cell respiration, (E) MitoSox fluorescence, following treatment for 5 d with dsRNA against the genes indicated. All data normalized to untreated cells on day zero. Means + SD from at least 4 experiments, each conducted in triplicate. Asterisks (*) indicate significant differences from control and from GFP values (p<0.01).
