# Supplementary material for: Screen for mitochondrial DNA copy number maintenance genes reveals essential role for ATP synthase
Source: Mol Syst Biol. 2014 Jul 1;10(6):734. doi: 10.15252/msb.20145117 (PMC4265055; doi:10.15252/msb.20145117)
Supplement: Supplementary file 3 — Supplementary Figure S3 [file msb0010-0734-sd3.pdf]

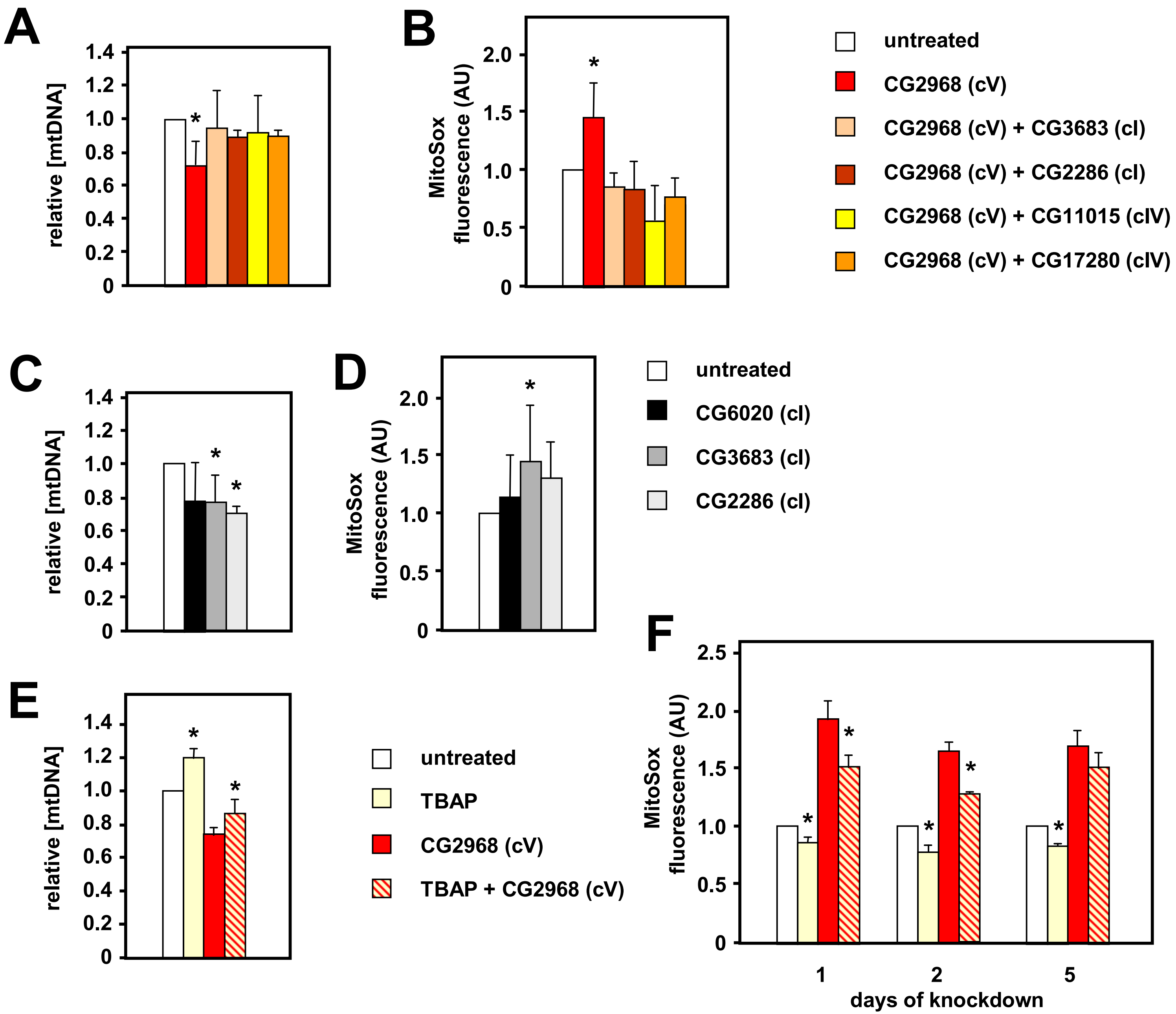

Figure S3, Fukuoh et al

Figure S3

Effects of various treatments on mtDNA copy number and turnover

(A, C, E) Relative mtDNA level and (B, D, F) MitoSox fluorescence, following treatment with dsRNA against the genes indicated, with or without treatment with TBAP, where shown. Knockdown was for 5 d except where indicated. Means ± SD from at least 4 independent experiments, each conducted in triplicate. Note that data for CG6020 from Fig. S2B, C are replotted to show comparison with knockdown of other cI genes. (A-D) Asterisks (*) indicate significant differences from untreated cell values (p<0.01, p<0.05 in panels C, D). (E, F) Asterisks (*) indicate significant differences from values for corresponding cells not treated with TBAP (p<0.05 or <0.022 after Bonferroni correction).
