# Supplementary material for: Screen for mitochondrial DNA copy number maintenance genes reveals essential role for ATP synthase
Source: Mol Syst Biol. 2014 Jul 1;10(6):734. doi: 10.15252/msb.20145117 (PMC4265055; doi:10.15252/msb.20145117)
Supplement: Supplementary file 4 — Supplementary Figure S4 [file msb0010-0734-sd4.pdf]

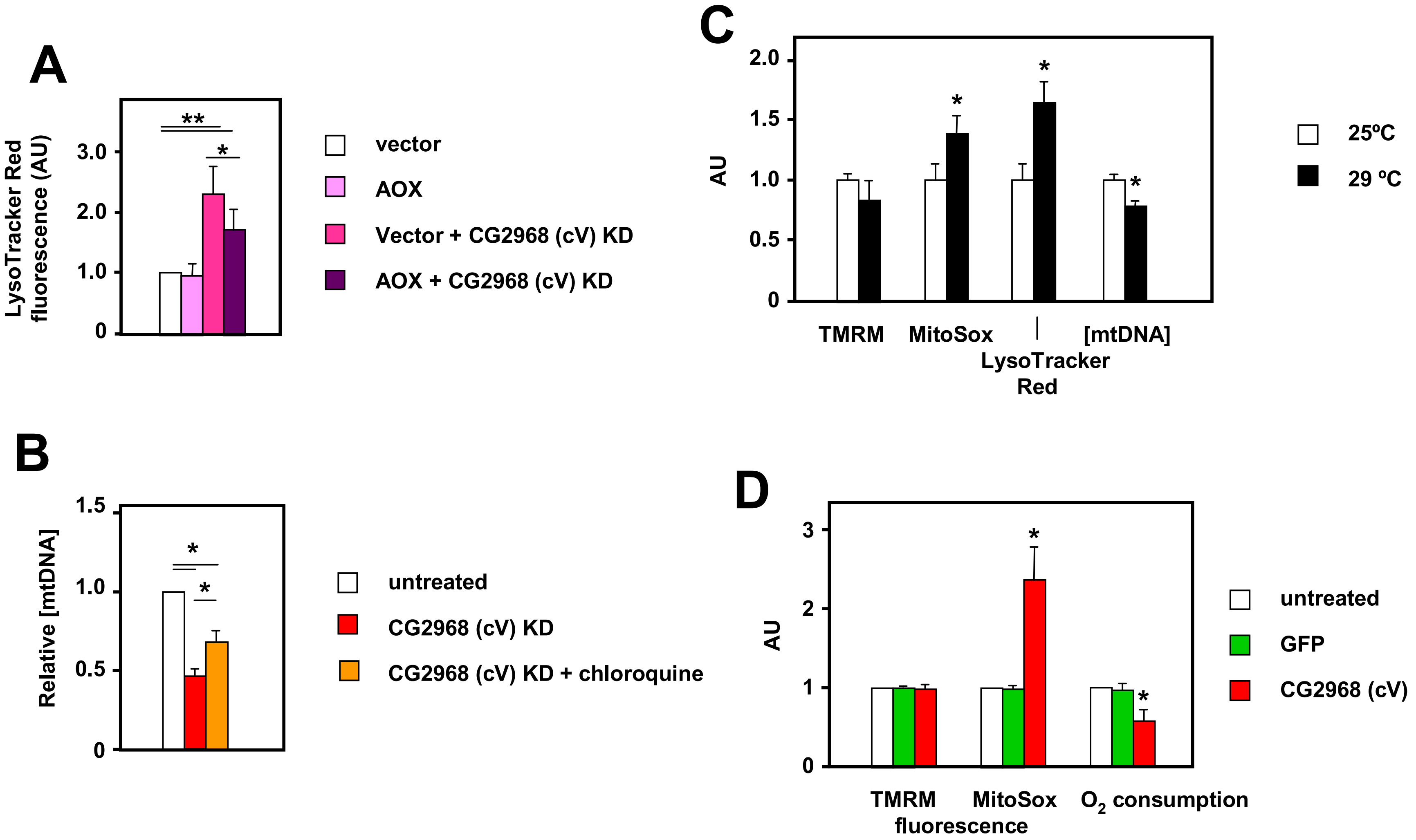

Figure S4, Fukuoh et al

Figure S4

Mitochondrial turnover and bioenergetic parameters during cV knockdown or heat stress

(A) LysoTracker Red fluorescence in cells with or without AOX expression or CG2968 knockdown, as indicated, normalized to that in untreated control cells. Means + SE from $\geq$ 4 replicate experiments. Asterisks denote statistical significance (* $p<0.05$, ** $p<0.01$, *t* test, Bonferroni corrected). (B) Effect of 100 μM chloroquine treatment on mtDNA copy number depletion effected by dsRNA against CG2968. Copy number normalized to value from untreated cells, as shown. Asterisks denote statistical significance ($p<0.01$, *t* test, Bonferroni corrected). (C) Relative TMRM, MitoSox and LysoTracker Red fluorescence and mtDNA copy number in *Drosophila* S2 cells cultured for 3 d at 25 ºC or 29 ºC, normalized to mean values for cells at 25 ºC. Means + SE, data from $\geq$3 replicate experiments. Asterisks denote statistical significance ($p<0.05$, *t* test). (D) TMRM and MitoSox fluorescence, and whole cell respiration, following 5 d of dsRNA treatment targeted against the genes indicated, corrected for mitochondrial mass by normalization to MitoTracker Green fluorescence, then normalized against values for control cells. Means + SD, data from $\geq$3 replicate experiments. Asterisks denote statistical significance ($p<0.01$, *t* test, Bonferroni corrected).
