# Supplementary material for: Screen for mitochondrial DNA copy number maintenance genes reveals essential role for ATP synthase
Source: Mol Syst Biol. 2014 Jul 1;10(6):734. doi: 10.15252/msb.20145117 (PMC4265055; doi:10.15252/msb.20145117)
Supplement: Supplementary file 5 — Supplementary Figure S5 [file msb0010-0734-sd5.pdf]

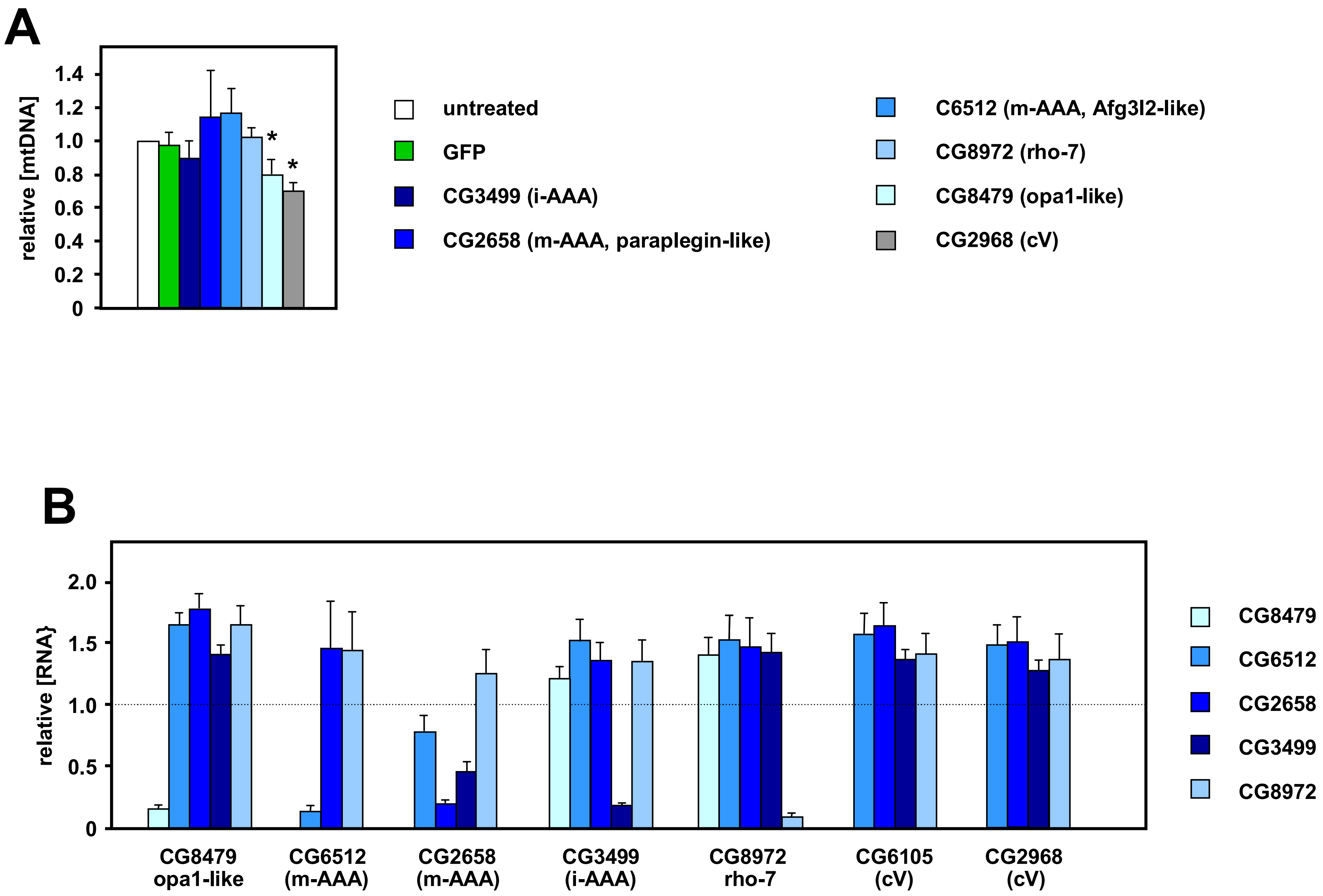

Figure S5, Fukuoh et al

Figure S5

Effects of knockdown of *opa1-like* and various mitochondrial proteases

(A) mtDNA copy number after 5 d of dsRNA treatment targeted against the genes indicated, normalized to value for untreated cells grown in parallel. (B) mRNA levels of the genes indicated, after 5d of dsRNA treatment targeted against the genes shown below each set of bars, based on QRTPCR normalized to the value for untreated cells grown in parallel. For clarity, data is only shown for those mRNAs whose levels were significantly different from 1 at the level $p<0.01$, and which showed a proportionate change from untreated cells of >20%. Means $\pm$ SD from at least 3 independent experiments, each conducted in triplicate.
