# Supplementary material for: Screen for mitochondrial DNA copy number maintenance genes reveals essential role for ATP synthase
Source: Mol Syst Biol. 2014 Jul 1;10(6):734. doi: 10.15252/msb.20145117 (PMC4265055; doi:10.15252/msb.20145117)
Supplement: Supplementary file 13 — Supplementary Materials and Methods [file msb0010-0734-sd13.pdf]

SUPPLEMENTARY MATERIALS AND METHODS

Cell maintenance

Drosophila S2 cells (Invitrogen) were grown in plastic flasks at 25 ºC in Schneider´s insect medium (Sigma) supplemented with 10% FBS, 100 U/ml of penicillin/streptomycin (Lonza) and diluted 1:6 into fresh medium every 3-4 d. In specific experiments glucose in the medium was replaced with the same concentration of galactose. S2 cells stably expressing *Ciona intestinalis* AOX were generated by cotransfection of 5×10^5 cells with pCoHygro (Invitrogen) and pAC/AOX, containing the AOX cDNA (Hakkaart et al, 2006) under the control of a *Drosophila* β-actin promoter, using FuGENE® HD Transfection Reagent (Promega) according to manufacturer's instructions. After 48 h of normal culture, a clone of AOX-transfected cells was selected using 300 ng/ml of Hygromycin B. For testing the effect of oligomycin, S2 cells were seeded into 6-well plates (2 ml, 10^6 cells/ml), allowed to attach for 30 min at 25˚C, and oligomycin was added to 50 nM. Cells were harvested after 5 d and incubated for MitoSox fluorescence or processed for DNA isolation as described below.

Screening of *Drosophila* dsRNA library in a 96-well plate format

S2 cells were seeded into 96-well plates (0.1 ml, 10^6 cells/ml) and allowed to attach for 30 min at 25˚C, after which the medium was replaced with fresh medium containing 0.6-1.2 μg of dsRNA from the dsRNA library (Open BioSystems, 16,016 dsRNAs targeting >13,000 annotated protein-coding genes). In each 96-well plate, 0.8 μg of dsRNA against positive and negative controls was added, prepared as described previously (Jõers et al, 2013), by transcription pf customized PCR products with T7 RNA polymerase. Each primer used in the PCR contained a 5′ end sequence corresponding to the T7 polymerase promoter (GAATTAATACGACTCACTATAGGGAGA), followed by a gene-specific sequence portion. *tamas* (CG8987), mtDNA helicase (CG5924) and *mTTF* (CG18124), were used as positive controls (see Table S6 for primer sequences) and an inert dsRNA against GFP was used as a negative control (Jõers et al, 2013). After culture for 3 d at 25 ˚C, 0.8 μg of dsRNA

targeted against TFAM was added to each well, and cells cultured for a further 2 d at 25˚C. In test experiments, spiking the dsRNA in this way for 48 h produced no significant additional drop in mtDNA copy number, but did enable mtDNA nucleoids to be much more clearly visualized in control cells, presumably because of the documented effects of TFAM on mtDNA topology (Pohjoismäki et al, 2006). Note also that spiking against TFAM was not used in any experiments in which mtDNA copy number, RNA levels or bioenergetic parameters were analysed. The high knockdown efficiency using this procedure in S2 cells has previously been documented (Clemens et al, 2000; Kleino et al, 2005). In the course of follow-up work, knockdown was also verified at the RNA level by QRTPCR for 17 specific targets, giving knockdown of $\geq$75% for CG2658, CG2968, CG3499, CG4217, CG4644, CG5047, CG5924, CG6020, CG6512, CG7175, CG8479, CG8972, CG8987, CG15390 and CG18124, and knockdown of ~40% and ~50%, respectively (Fig. S2), for the remaining two targets CG6105 and CG9032.

Fluorescence microscopy of mtDNA nucleoids

Nucleoids were visualized after cells were transferred to a fresh 96-well plate, and allowed to attach for 30 min at 25 ˚C. The medium was then replaced with medium containing Quant-iT™ PicoGreen® dsDNA reagent (7.5 µl/ml, Invitrogen) and cells were incubated for 30 min at 25˚C. After two rinses with normal medium, cells were incubated for a further 2 h in 0.1 ml of normal medium, rinsed once more with 0.1 ml of medium to remove debris, and imaged by fluorescence microscopy (OLYMPUS IX71 with QCapture Pro software, Olympus). Images were scored manually (see main text). A computational method which we developed to judge the intensity of punctate fluorescence signals in the captured images (details to be published elsewhere) gave many false positives, due to variability of background fluorescence, as well as false negatives due to signal from cell debris. More specifically, with thresholds set to capture 79 of the 105 manually defined positives, over 2500 other dsRNAs were scored as positive by the software, most of them clearly artefactual to the human eye, whilst 13 of the 25 manual positives that were missed by the software proved repeatedly positive upon

rescreening. Using a lower threshold, which identified only half of the ‘manual’ positives, 400 other positives were picked out by the computational method. Most of which we judged as artefacts. Based on repeated rescreening, in which 86 of the 105 original positives were retained (including splice variants), we judged the software to be inferior to subjective judgement, at least in identifying the major classes of positives, which was the main purpose of the screen. Criteria for assigning definitive positives were established, as described in the main text. Images for all 97 definitive positives are shown in Figure S1.

dsRNA treatment for analysis of cellular parameters

For larger-scale dsRNA treatment, 1.5µg of total RNA was reverse-transcribed to cDNA using RT random primers and MultiScribe Reverse Transcriptase (Applied Biosystems), according to manufacturer’s instructions. dsRNAs were prepared as above, except that amplification conditions were varied according to the predicted melting temperatures of the primers used (see Table S5 for primer sequences). The PCR products were separated on 1% agarose gels, excised and purified by the NucleoSpin ExtractII System (Biotop), then used as templates for the MEGAscript T7 transcription kit (Ambion) according to manufacturer's instructions. The dsRNA obtained was treated with 1µl of TURBO DNase for 15 min at 37 ºC, purified by phenol-chloroform extraction, precipitated by adding 1 vol of isopropanol, resuspended in DEPC-treated water and the relative concentration determined using a Nanodrop^{2000} spectrophotometer (Thermo Scientific). For initial mtDNA copy number analysis, 5 x 10^5 S2 cells in 0.5 ml of medium were seeded per well of a 24-well plate, with addition of 4 µg of dsRNA after cell attachment, and a further 4 µg on day 3. Cells were harvested after 5 d. For other experiments S2 cells were diluted to a final concentration of 1.25 x 10^5 cells/ml in 2 ml of complete *Drosophila* medium in 6-well plates. After 30 min, 4 µg of dsRNA was added directly to the medium. Cells were swirled by hand, incubated at 25°C and at day 3 new dsRNA was added. For testing the effect of chloroquine, S2 cells were seeded into 12-well plates (1 ml, 1.5 x 10^5 cells/ml) in medium containing 100 µM

chloroquine, with addition of 4 µg of dsRNA after cell attachment, plus a further 4 µg on day 3, and harvested after 5 d.

RNA and DNA isolation

Total RNA was isolated from S2 cells as described previously (Jõers et al, 2013). For DNA isolation for initial copy-number analysis, cells from a single well of a 24-well plate, treated as described above, were centrifuged for 3 min at 1200 g_{max}, and washed in 700 µl PBS. Cells were finally resuspended in 500 µl cell lysis buffer (75 mM NaCl, 50 mM EDTA, 20 mM HEPES pH 7.8), to which was added 5 µl of 20% SDS. After mixing by inverting the tube until the solution became clear, 10 µl of proteinase K (20 mg/ml, Fermentas) was added, with further mixing, followed by incubation for 2 h at 50 °C. DNA was precipitated overnight at -20 ° by the addition of 412 µl isopropanol. Samples were equilibrated at 4 °C for 1 h, centrifuged for at 4 °C and 16000 g_{max} for 30 min, and washed with 1 ml of ice-cold 70% ethanol. DNA pellets were air-dried and resuspended in 100 µl of (10 mM Tris/HCl, 0.1 mM EDTA, pH 7.5) and incubated overnight at 55 °C to properly dissolve the DNA. For follow-up experiments, DNA was prepared from 1.5 x 10^6 cells cultured in 6-well plates, as previously (Jõers et al, 2013).

Quantitative PCR

Mitochondrial DNA copy number was assessed by QPCR using primers against COXII (for mtDNA) and RpL32 (nuclear DNA, single-copy, for normalization: see Table S6 for primer sequences). The reaction mix for each sample (20 µl) contained 10 pmol each of forward and reverse primers, 10 µl SYBR green mix (Applied Biosystems) and 5 ng total DNA. Reactions were run on the StepOnePlus™ Real-Time PCR System (Life Technologies) with cycle conditions 95°C for 20 s, 40 cycles of 95°C for 3 s and 60°C for 30 s, followed by a melt curve stage. Transcript levels of CG3499, CG6512, CG2658, CG8972 and CG8479 in S2 cells were estimated relative to that of RpL32 using a similar procedure, except that the template was 1 µl of a cDNA reaction, diluted 1:100 in water. For primers, see Table S7.

Measurements of mitochondrial function

Following dsRNA or other treatments, 2 x 10^5 cells were incubated with cell-permeant fluorescent indicators of mitochondrial membrane potential or ROS, respectively 200 nM tetramethylrhodamine methyl ester (TMRM) for 30 min at 25 °C or 2.5 µM MitoSox^{TM} (Invitrogen) for 45 min at 25 °C or, for mitochondrial mass measurement, 200 nM 10-nonyl acridine orange (NAO) or 40 nM MitoTracker® Green FM (Life Technologies) for 30 min at 25 °C. Medium was replaced with PBS and cells were kept at room temperature for TMRM, NAO and MitoTracker Green staining or on ice for MitoSox, before flow cytometry analysis (Cannino et al, 2012). Cell fluorescence was counted from 40,000 objects. The region of interest was defined using forward scatter/side scatter values, excluding debris and dead cells. Cell fluorescence was measured with an accuri-C6 flow cytometer (488 nm excitation) using the FL1 channel (516 nm emission) for MitoTracker Green, the FL2 channel, (585 ±40 nm emission) for TMRM and NAO or the FL3 channel (>670 nm emission) for MitoSox. Oxygen consumption by living cells was measured in a Clark-type electrode chamber (Hansatech Oxyterm system) using 5 x 10^6 cells (41). dsRNA-treated or controls cells were suspended in 0.55 ml of culture medium in the oxygraph chamber and used immediately for O_2 consumption measurements. Total cellular respiration was corrected for non-respiratory oxygen consumption, defined as the component of oxygen consumption in the presence of rotenone (150 nM) and potassium cyanide (200 nM).

Analyses of lysosomal and mitochondrial content

Following dsRNA or chloroquine treatment (50 µM for 24 h) cells were incubated for 10 min at 25°C in prewarmed medium containing 50 nM LysoTracker Red DND-99 (Life Technologies). Cells were then resuspended in fresh prewarmed medium and red lysosomal fluorescence of 40,000 cells per sample was determined as above, by flow cytometry using the FL3 channel. For imaging of mitochondria and lysosomes in living cells, 5 x 10^5 cells were resuspended in 1.5 ml of Schneider S2 medium containing 25 nM MitoTracker Green

FM and seeded on glass-bottomed microwell dishes (MatTek) coated with Concanavalin A (0.5 mg/ml) as described previously (Rogers et al, 2008). After 45 min of incubation at 25 °C cells were washed three times with medium, after which LysoTracker Red DND-99 (Life Technologies) was added to 50 nM, with further incubation for 5 min. Images were captured using a Nikon Eclipse Ti inverted Microscope, Andor camera, spinning disk confocal system, and Andor IQ2 software. Filters used were 488 nm for MitoTracker Green and 568 nm for LysoTracker Red. For spot-area calculation, microscopy images from a single plane or from z stacks were analyzed, measured and processed by ImageJ software, a public domain Java Image processing program, and data were further processed by Microsoft Excel™. For deconvolution, multi-channel image stacks were separated into single-channel stacks (Fiji, open-source) and deconvolved with Huygens software (SVI), with microscope and objective parameters as follows: numerical aperture: 1.4, refractive index: 1.515, media refractive index: 1.338, pinhole back-projection 2,530 nm. Sample density in X, Y and Z axes was, respectively, 89.5, 89.5 and 196.1 nm. Excitation and emission wavelengths for MitoTracker Green- and LysoTracker Red-stained images, respectively, were set to 490/516 nm and 577/590 nm. In both cases the pinhole back-projection radius was calculated at 250 nm. Batch deconvolutions were performed using automatic background correction, an estimated signal to noise ratio of 15, 300 maximum iterations and a quality threshold of 1%. Separate channels were reassembled into an RGB image in Photoshop; transferring, for the selected image of a stack, the MitoTracker deconvolved picture to the green channel and the LysoTracker deconvolved one to the red channel. Channel intensities were adjusted for best resolution.

Western blotting

Post-nuclear extracts were prepared from 10^6cells washed in PBS, pelleted and suspended in lysis buffer containing 50 mM Tris/Hcl pH 7.4, 150 mM NaCl, 1 mM EDTA, 1% Triton X-100 and a protease inhibitor cocktail (Roche). The samples were incubated for 30 min on ice and centrifuged at 14,000 g_{max} for 5 min, after which the supernatant was collected and protein concentration measured using the Bradford assay. The extracts were electrophoresed

on a denaturing SDS-12.5% polyacrylamide slab gel and blotted onto a Hybond-C Extra nitrocellulose membrane (Amersham Biosciences) in transfer buffer containing 25 mM Tris, 200 mM glycine, 20% methanol and 0.02% SDS (pH 8.2) at a constant voltage of 80 V for 45 min at 4 ºC. Membranes were incubated in blocking solution containing 5% nonfat milk in TBST (10 mM Tris/HCl pH 8.0, 150 mM NaCl, 0.05% Tween 20) for 1 h at room temperature with gentle shaking and then with primary antibody in TBST containing 5% nonfat milk overnight at 4 ºC with gentle shaking. Antibodies used were against NDUFS3 (Abcam 14711, mouse monoclonal, 1:10,000), ATP5A (Abcam 14748, mouse monoclonal, 1:1,000) and GAPDH (C Terminus, Everest Biotech 06377, goat polyclonal, 1:2,000). Membranes were washed three times for 10 min in TBST and incubated with a 1:10,000 dilution of horseradish peroxidase-conjugated secondary antibodies (horse anti-mouse, Vector Laboratories PI-2000, 1:10,000, or donkey anti-goat, Santa Cruz sc-2020, 1:10,000) for 1 h in TBST containing 5% nonfat milk. Blots were washed with TBST three times and developed with the ECL system (Amersham Biosciences) according to the manufacturer's protocols.

Statistical analyses

Comparisons between populations were performed after testing for equality of the variances (F-test) followed by unpaired two-tailed Student's t tests, corrected for variance inequality where appropriate. Analyses of variance were used when more than two samples were compared. When analyses of variance indicated significant differences, a post-hoc multiple unpaired two-tailed Student's *t* test was performed, Bonferroni corrected for multiple comparisons where appropriate. All values are presented in graphs as means ± SE except where indicated.

REFERENCES

Cannino G, El-Khoury R, Pirinen M, Hutz B, Rustin P, Jacobs HT, Dufour E (2012) Glucose modulates respiratory complex I activity in response to acute mitochondrial dysfunction. *J Biol Chem* **287**: 38729-38740.

Clemens JC, Worby CA, Simonson-Leff N, Muda M, Maehama T, Hemmings BA, Dixon JE (2000) Use of double-stranded RNA interference in *Drosophila* cell lines to dissect signal transduction pathways. *Proc Natl Acad Sci USA* **97**: 6499-6503.

Hakkaart GA, Dassa EP, Jacobs HT, Rustin P (2006) Allotopic expression of a mitochondrial alternative oxidase confers cyanide resistance to human cell respiration. *EMBO Rep* **7**: 341-345.

Kleino A, Valanne S, Ulvila J, Kallio J, Myllymäki H, Enwald H, Stöven S, Poidevin M, Ueda R, Hultmark D, Lemaitre B, Rämet M (2005) Inhibitor of apoptosis 2 and TAK1-binding protein are components of the *Drosophila Imd* pathway. *EMBO J* **24**: 3423-3434.

Jõers P, Lewis SC, Fukuoh A, Parhiala M, Ellilä S, Holt IJ, Jacobs HT (2013) Mitochondrial transcription terminator family members mTTF and mTerf5 have opposing roles in coordination of mtDNA synthesis. *PLoS Genet* **9**: e1003800.

Pohjoismäki JL, Wanrooij S, Hyvärinen AK, Goffart S, Holt IJ, Spelbrink JN, Jacobs HT (2006) Alterations to the expression level of mitochondrial transcription factor A, TFAM, modify the mode of mitochondrial DNA replication in cultured human cells. *Nucleic Acids Res* **34**: 5815-5828.

Rogers SL, Rogers GC (2008) Culture of Drosophila S2 cells and their use for RNAi-mediated loss-of-function studies and immunofluorescence microscopy. *Nat Protoc* **3**: 606-611.
